# Clinical features analysis for complications in infants with late-onset Group B streptococcal sepsis: a retrospective case-control study

**DOI:** 10.3389/fmed.2025.1657655

**Published:** 2025-11-13

**Authors:** Haifeng Geng, Wenqiang Sun, Qiuping Shen, Cancan Li, Wenmei Li, Huawei Wang, Zhixin Wu, Xueping Zhu

**Affiliations:** Department of Neonatology, Children's Hospital of Soochow University, Suzhou, China

**Keywords:** infants, Group B streptococcus, late-onset, sepsis, complications, risk factors

## Abstract

**Objective:**

To investigate the risk factors associated with complications in infants with late-onset Group B Streptococcal (GBS) sepsis, and to provide evidence for clinical intervention strategies.

**Methods:**

This study is a retrospective case-control study. The clinical data of 101 infants with late-onset GBS sepsis, diagnosed before 3 months of age were retrospectively analyzed. According to the presence or absence of complications, the infants were divided into the complication group and the non-complication group. Univariate and multivariate analyses were performed to explore the risk factors associated with the occurrence of complications in infants with late-onset GBS sepsis. Using ROC curves to evaluate the predictive efficacy of clinical variables.

**Results:**

A total of 101 cases of late-onset GBS sepsis met the inclusion criteria, including 41 in the non-complication group and 60 in the complication group. The gestational age in the complication group was significantly lower than that in the non-complication group (*P* < 0.05). Clinically, the complication group had a higher incidence of seizure, bulging anterior fontanelle, and fever, as well as a significantly lower PaO_2_/FiO_2_ ratio (*P* < 0.05). Laboratory findings showed that the complication group had a higher incidence of hypoalbuminemia, concomitant positivity in blood and cerebrospinal fluid cultures, elevated creatinine and blood urea nitrogen levels, and significantly lower pH and albumin levels (*P* < 0.05). In addition, the complication group exhibited significantly higher Pediatric Sequential Organ Failure Assessment (pSOFA) scores, Pediatric Logistic Organ Dysfunction Score 2 (PELOD-2) scores, and higher proportion of patients with high pSOFA (>3.50) and PELOD-2 scores (>3.50) (*P* < 0.05). Multivariate analysis revealed that high PELOD-2 score, high pSOFA score, high creatinine levels and hypoalbuminemia were risk factors for the development of complications in infants with late-onset GBS sepsis; the ROC curve constructed using these predictors demonstrated excellent discriminative ability, with an AUC of 0.858 (95% CI: 0.782–0.934), sensitivity of 77.78%, and specificity of 82.61%.

**Conclusion:**

High PELOD-2 score, high pSOFA score, high creatinine levels, and hypoalbuminemia independently predict complications in late-onset GBS sepsis infants, enabling early risk stratification and tailored treatment to improve outcomes.

## 1 Introduction

Group B Streptococcal (GBS) sepsis is a serious life-threatening condition in neonates, often leading to multi-organ dysfunction. It is frequently associated with complications such as meningitis, septic shock, disseminated intravascular coagulation (DIC), pyogenic arthritis, and osteomyelitis. These complications not only increase mortality but can also result in long-term sequelae, including neurodevelopmental impairments and skeletal abnormalities, severely affecting quality of life (1, 2). Although significant progress has been made in the prevention and treatment of neonatal GBS infections due to the widespread use of antibiotics and the implementation of prenatal screening, the clinical presentation of late-onset GBS sepsis remains variable. Symptoms often emerge during the middle or late stages of the disease course, contributing to a high incidence of complications and mortality (2–4).

Late-onset GBS sepsis typically occurs between 7 and 90 days after birth and is characterized by clinical manifestations of multi-organ dysfunction following infection. Existing studies have identified several potential risk factors for GBS sepsis, including gestational age, birth weight, physiological status at the time of infection, and the maturity of the immune system (5, 6). However, the specific risk factors associated with complications in infants with late-onset GBS sepsis remain incompletely understood. Current literature lacks comprehensive multivariate analyses, resulting in gaps in the clinical assessment and management of this condition. Identifying the risk factors for complications in late-onset GBS sepsis is of great significance for optimizing clinical prevention and treatment strategies and improving patient outcomes.

Therefore, this study aimed to retrospectively analyze the clinical data of infants under 3 months of age with late-onset GBS sepsis who were admitted to our hospital between January 1, 2007, and December 31, 2022. The objective was to identify independent risk factors associated with the development of complications, in order to provide valuable predictive indicators and intervention strategies for clinical practice. These findings may contribute to improving the clinical management of infants with late-onset GBS sepsis, reducing the incidence of complications, and enhancing patient outcomes.

## 2 Materials and methods

### 2.1 Study subjects and design

This study was designed as a retrospective case-control study. Infants under 3 months of age diagnosed with late-onset GBS sepsis and admitted to the Children's Hospital of Soochow University between January 1, 2007, and December 31, 2022, were included as study subjects. Infants with severe congenital malformations and/or inherited metabolic disorders were excluded. This study adheres to the ethical standards approved by the Medical Ethics Committee of the Children's Hospital of Soochow University (Approval No. 2022CS010). All data were anonymized, and no biological samples or personally identifiable information were involved. The requirement for informed consent from the guardians was waived with the approval of the Ethics Committee. All study procedures were performed according to the ethical standards in the Declaration of Helsinki.

### 2.2 Grouping, diagnostic criteria, and related definitions

Patients were divided into a complication group and a non-complication group based on the presence or absence of sepsis-related complications. The diagnostic criteria for late-onset GBS sepsis and associated complications were based on previously published standards (7). Specifically, the diagnosis required the presence of relevant clinical signs and symptoms plus microbiological confirmation via a positive GBS culture obtained from a sterile body cavity (e.g., blood or cerebrospinal fluid). Late-onset GBS sepsis was defined as GBS sepsis with an onset age of ≥7 days.

Complications primarily included septic shock, disseminated intravascular coagulation (DIC), primary or metastatic suppurative inflammation or abscesses (such as hepatic abscess, pericarditis, purulent meningitis, septic arthritis, and osteomyelitis). Septic shock: sepsis-induced circulatory and tissue hypoperfusion with persistent hypotension requiring vasoactive support to maintain adequate mean arterial pressure (e.g., dopamine >5 μg/kg·min or any dose of dobutamine, norepinephrine, or epinephrine), together with clinical or laboratory evidence of hypoperfusion. GBS-related purulent meningitis is characterized by clinical symptoms of purulent meningitis, CSF findings consistent with the diagnosis, and the isolation of group *B Streptococcus* from CSF and/or blood cultures. DIC: an acquired coagulopathy characterized by systemic activation of coagulation leading to widespread microvascular thrombosis, consumptive depletion of coagulation factors and platelets, and secondary hyperfibrinolysis, presenting with bleeding tendency and microcirculatory failure. Osteomyelitis: diagnosis is based on compatible clinical features (limb/joint pain with fever), supportive laboratory abnormalities (elevated ESR and leukocytosis), imaging evidence of deep soft-tissue swelling and/or bony destruction, and confirmation of infection by aspiration or operative drainage of pus. Final diagnosis integrates clinical, laboratory, imaging, and microbiological data. All the above diagnoses are made with reference to *Zhu Futang Practice of Pediatrics* and *Practical Neonatology* (7, 8). Abnormal peripheral white blood cell count (including leukocytosis and leukopenia), thrombocytopenia, and pathological jaundice were defined according to the criteria outlined in *Zhu Futang Practice of Pediatrics* and *Practical Neonatology* (7, 8). Neonatal hypoalbuminemia was defined as a serum albumin level of < 25 g/L (9).

### 2.3 Data collection

All clinical data were retrieved from the hospital's electronic medical record system. Information collected included the infant's basic characteristics (sex, birth weight, age at admission), perinatal factors (gestational age at delivery, mode of delivery, presence of clinical chorioamnionitis, and premature rupture of membranes), initial clinical manifestations, and laboratory findings within 24 h of admission. Laboratory evaluations encompassed white blood cell (WBC) count, platelet count, C-reactive protein (CRP), procalcitonin (PCT), liver and renal function tests, coagulation profile, arterial blood gas analysis, blood culture, and cerebrospinal fluid (CSF) examination.

### 2.4 Score the scale

The Pediatric Sequential Organ Failure Assessment (pSOFA) score (10): was calculated on the first day of admission for all infants. The pSOFA score evaluates six organ systems: respiratory, coagulation, liver, cardiovascular, neurological, and renal, with each system scored from 0 to 4. The total pSOFA score was obtained by summing the individual scores of each system on day 1 of admission. The Pediatric Logistic Organ Dysfunction Score 2 (PELOD-2) (11): was used to assess dysfunction in five systems: central nervous, cardiovascular, renal, respiratory, and hematologic. Each system is scored from 0 to 6, and the total PELOD-2 score was calculated by summing the individual system scores on the first day of admission (for infants transferred from another hospital, scoring was based on the first admission day at the referring hospital). A high pSOFA/PELOD-2 score was defined as a score exceeding the optimal cutoff value determined by the receiver operating characteristic (ROC) curve analysis for predicting the occurrence of complications in infants with late-onset GBS sepsis in this study.

### 2.5 Statistical analysis

The data were analyzed using SPSS version 26.0. For continuous variables with a normal distribution, results were expressed as x̄ ±*s*, and comparisons between groups were performed using the independent samples *t*-test. For non-normally distributed continuous variables, data were presented as median (P_25_-P_75_) and compared using the Mann–Whitney U-test. Categorical variables were expressed as frequencies and percentages, and group comparisons were conducted using the χ^2^ test. Multivariate logistic regression analysis was conducted to identify independent risk factors for complications. Construct the ROC curve to evaluate the predictive efficacy of clinical variables for the complications. All clinical variables considered as potential predictors were recorded preceding the diagnosis of the adverse outcome. A *P*-*value* < 0.05 was considered statistically significant.

## 3 Results

Between 2007 and 2022, a total of 199 infants with GBS sepsis were hospitalized and treated at our institution, among whom 101 cases (50.75%) were classified as late-onset GBS sepsis. Among these, 41 infants were assigned to the non-complication group and 60 to the complication group. Within the complication group, there were 4 cases of septic shock, 44 cases of purulent meningitis, 1 case of osteomyelitis, 3 cases of septic shock with DIC, 5 cases of purulent meningitis combined with septic shock, and 3 cases of purulent meningitis combined with both septic shock and DIC.

### 3.1 General characteristics

The general characteristics of the two groups are summarized in Table 1. There were no significant differences between the complication and non-complication groups in terms of sex, prematurity, birth weight, small for gestational age, maternal age, fever before delivery, mode of delivery, chorioamnionitis, meconium-stained amniotic fluid, asphyxia at birth, premature rupture of membranes ≥18 h, or breastfeeding (*P* > 0.05). However, the gestational age was significantly lower in the complication group compared to the non-complication group (*P* < 0.05).

**Table 1:** Baseline characteristics of late-onset GBS sepsis infants.

**Characteristics**	**Non-complication group (*n* = 41)**	**Complication group (*n* = 60)**	***P*-value**
Male [*n* (%)]	18 (43.90)	29 (48.33)	0.802
Premature birth [*n* (%)]	1 (2.44)	9 (15.00)	0.094
Birth weight [x̄ ± s, g]	3299.25 ± 380.347	3152.62 ± 402.127	0.577
Gestational age [M (P25, P75), weeks]	39.40 (39.10, 40.10)	39.00 (37.30, 39.40)	0.001
SGA [*n* (%)]	2 (4.88)	4 (6.67)	1.000
Maternal age [M (P25, P75), years]	25.50 (23.00, 31.00)	25.00 (22.00, 27.50)	0.101
Fever before delivery [*n* (%)]	3 (7.32)	5 (8.33)	1.000
Vaginal delivery [*n* (%)]	29 (70.73)	39 (65.00)	0.369
Chorioamnionitis [*n* (%)]	4 (9.76)	8 (13.33)	0.874
MSAF [*n* (%)]	4 (9.76)	7 (11.67)	1.000
Asphyxiation at birth [*n* (%)]	3 (7.32)	5 (8.33)	1.000
PROM ≥18 h [*n* (%)]	3 (7.32)	6 (10.00)	0.963
Breast feeding [*n* (%)]	26 (63.41)	42 (70.00)	0.686

GBS, Group B Streptococcal; SGA, small for gestational age; MSAF, Meconium-stained amniotic fluid; PROM, Premature rupture of membranes.

### 3.2 Clinical manifestations and vital signs at admission

The clinical manifestations of the two groups are presented in Table 2. Compared with the non-complication group, the complication group had significantly higher incidences of seizure and bulging anterior fontanelle, as well as higher fever temperatures and lower PaO_2_/FiO_2_ ratios (*P* < 0.05). There were no significant differences between the two groups in the proportions of patients presenting with fever, decreased responsiveness, jaundice, tachypnea, poor feeding, somnolence, irritability, muscle tone abnormalities, mottling of the skin, cyanosis, and vomiting (*P* > 0.05).

**Table 2:** Clinical manifestation in late-onset GBS sepsis infants.

**Characteristics**	**Non-complication group (*n* = 41)**	**Complication group (*n* = 60)**	***P*-value**
**Clinical manifestation**			
Fever [*n* (%)]	34 (82.93)	51 (85.00)	0.851
Decreased responsiveness [*n* (%)]	22 (53.66)	42 (70.00)	0.158
Jaundice [*n* (%)]	3 (7.32)	7 (11.67)	0.754
Tachypnea [*n* (%)]	14 (34.15)	19 (31.67)	0.686
Poor feeding [*n* (%)]	18 (43.90)	39 (65.00)	0.061
Somnolence [*n* (%)]	19 (46.34)	34 (56.67)	0.417
Seizure [*n* (%)]	3 (7.32)	27 (45.00)	0.000
Irritability [*n* (%)]	8 (19.51)	23 (38.33)	0.059
Bulging anterior fontanelle [*n* (%)]	1 (2.44)	13 (21.67)	0.007
Muscle tone abnormalities [*n* (%)]	4 (9.76)	11 (18.33)	0.288
Mottling of the skin [*n* (%)]	11 (26.83)	21 (35.00)	0.643
Cyanosis [*n* (%)]	11 (26.83)	19 (31.67)	0.695
Vomiting [*n* (%)]	4 (9.76)	13 (21.67)	0.137
**Vital signs**			
Temperature [M (P25, P75),°C]	38.90 (37.80, 39.10)	39.00 (38.55, 39.75)	0.010
Respiration [M (P25, P75), beats/min]	43.5 (40, 59.5)	45 (42,53.50)	0.181
Heart rate [x̄ ± s, beats/min]	134.28 ± 17.54	136.95 ± 19.30	0.482
PaO2/FiO2 [M (P25, P75), mmHg]	402.5 (369.75, 422.00)	378.00 (253.00, 405.00)	0.006

GBS, Group B Streptococcal.

### 3.3 Laboratory findings and critical illness scores at admission

Laboratory findings and severity scores within 24 h of admission are presented in Table 3. Compared with the non-complication group, the complication group showed significantly higher rates of hypoalbuminemia, concurrent positive blood and CSF cultures, elevated creatinine and blood urea nitrogen levels, higher pSOFA and PELOD-2 scores, and greater proportions of patients with high pSOFA and high PELOD-2 scores. In contrast, pH and serum albumin levels were significantly lower in the complication group (*P* < 0.05). There were no significant differences between the two groups in terms of WBC count, leukocytosis, leukopenia, platelet count, thrombocytopenia, PCT, CRP, fibrinogen, serum sodium, serum potassium, or lactic acid levels (*P* > 0.05).

**Table 3:** Laboratory tests and critical illness scores in late-onset GBS sepsis infants.

**Characteristics**	**Non-complication group (*n* = 41)**	**Complication group (*n* = 60)**	***P*-value**
WBC count [x̄ ± s, × 10^9^/L]	15.68 ± 8.22	15.11 ± 9.31	0.752
Leukocytosis [*n* (%)]	18 (43.90)	31 (51.67)	0.567
Leukopenia [*n* (%)]	5 (12.20)	10 (16.67)	0.590
Platelet count [x̄ ± s, × 10^9^/L]	329.88 ± 123.26	297.93 ± 130.26	0.221
Thrombocytopenia [*n* (%)]	2 (4.88)	5 (8.33)	0.827
Procalcitonin [M (P25, P75), ng/mL]	6.92 (3.75, 17.00)	10.00 (2.90, 19.40)	0.383
C-reactive protein [M (P25, P75), mg/L]	25.58 (13.40, 35.33)	34 (18, 50.73)	0.178
Fibrinogen [x̄ ± s, g/L]	2.93 ± 1.17	2.54 ± 1.17	0.109
Creatinine [x̄ ± s, mg/dl]	0.72 ± 0.15	0.87 ± 0.23	0.000
Urea nitrogen [x̄ ± s, mmol/L]	3.43 ± 0.58	3.66 ± 0.52	0.042
PH [x̄ ± s]	7.36 ± 0.10	7.31 ± 0.15	0.044
Serum Sodium [x̄ ± s, mmol/L]	137.70 ± 4.39	135.73 ± 5.99	0.060
Serum potassium [x̄ ± s, mmol/L]	4.10 ± 0.50	3.96 ± 0.67	0.253
Albumin [x̄ ± s, g/L]	31.82 ± 3.93	29.87 ± 4.34	0.024
Hypoalbuminemia [*n* (%)]	1 (2.44)	12 (20.00)	0.012
Concomitant positivity in blood and CSF cultures [*n* (%)]	0 (0)	39 (65.00)	0.000
Lactic acid [M (P25, P75), mmol/L]	3.70 (3.00, 4.93)	4.40 (3.10, 6.00)	0.103
pSOFA score [M (P25, P75)]	2 (2, 3)	5 (3, 7)	0.000
High pSOFA score [*n* (%)]	9 (21.95)	45 (75.00)	0.000
PELOD-2score [M (P25, P75)]	2 (0.25, 3.00)	5 (2.50, 7.00)	0.000
High PELOD-2 score [*n* (%)]	10 (24.39)	42 (70.00)	0.000

GBS, Group B Streptococcal; WBC, White Blood Cell; CSF, cerebrospinal fluid; pSOFA, Pediatric Sequential Organ Failure Assessment; PELOD-2, Pediatric Logistic Organ Dysfunction Score 2.

ROC curve analysis revealed that the optimal cut-off value for both the pSOFA and PELOD-2 scores in predicting complications in infants with late-onset GBS sepsis was 3.50. The area under the curve (AUC) was 0.784 (95% CI: 0.700–0.860) for the pSOFA score and 0.751 (95% CI: 0.660–0.840) for the PELOD-2 score. At this cut-off, the sensitivity and specificity were 76.70% and 78.00% for pSOFA, and 73.30% and 75.60% for PELOD-2, respectively.

### 3.4 Analysis of risk factors for complications in late-onset GBS sepsis

Multivariate logistic regression was performed to identify independent predictors of complications in infants with late-onset GBS sepsis. Variables were selected a priori based on clinical relevance and significant intergroup differences, including creatinine, urea nitrogen, PH, albumin, hypoalbuminemia, high pSOFA score, and high PELOD-2 score. The analysis of 101 complete cases (60 events; EPV = 8.571) yielded a statistically significant model (Omnibus test: χ^2^ = 36.755, *P* < 0.001; −2 Log Likelihood = 71.376) with good calibration (Hosmer-Lemeshow: χ^2^ = 6.031, *P* = 0.644). The model's explanatory power was substantial (Nagelkerke *R*^2^ = 0.501), with an overall accuracy of 82.10%. The absence of multicollinearity was confirmed (all VIF < 3.00, Tolerance > 0.40). It was ultimately found that the following factors were identified as being independently associated with adverse complications in infants with late-onset GBS sepsis: high PELOD-2 score (OR: 3.615, 95% CI: 1.027–12.727, *P* = 0.045), high pSOFA score (OR: 9.354, 95% CI: 1.989–43.984, *P* = 0.005), high creatinine levels (> 0.78 mg/dL) (OR: 1.130, 95% CI: 1.053–1.213, *P* = 0.001) and hypoalbuminemia (OR: 1.486, 95% CI: 1.010–2.186, *P* = 0.044). Details are shown in Table 4.

**Table 4:** Multivariate logistic regression analysis of complications in late-onset GBS sepsis infants.

**Characteristics**	**Regression coefficient**	**Standard error**	**Wald statistic**	***P- value***	**OR**	**95%CI**	**Tolerance**	**VIF**
Creatinine, mg/dl	0.122	0.036	11.420	0.001	1.130	1.053–1.213	0.897	1.114
Urea nitrogen, mmol/L	0.982	0.860	1.304	0.253	2.671	0.495–14.419	0.898	1.114
PH	0.002	0.003	0.767	0.381	1.002	0.997–1.007	0.864	1.158
Albumin, g/L	0.010	0.033	0.101	0.750	0.990	0.928–1.055	0.896	1.117
Hypoalbuminemia	0.396	0.197	4.052	0.044	1.486	1.010–2.186	0.863	1.159
High pSOFA score	2.236	0.790	8.013	0.005	9.354	1.989–43.984	0.950	1.052
High PELOD-2 score	1.285	0.642	4.003	0.045	3.615	1.027–12.727	0.828	1.208

GBS, Group B Streptococcal; PELOD-2, Pediatric Logistic Organ Dysfunction Score 2; pSOFA, Pediatric Sequential Organ Failure Assessment. [Creatinine (mg/dL): The OR of 1.130 indicates that for each 1.00 mg/dL increase in serum creatinine, the odds of the outcome increase by 13.00% (95% CI: 1.053961.213)].

### 3.5 ROC curve construction

**Figure 1:**
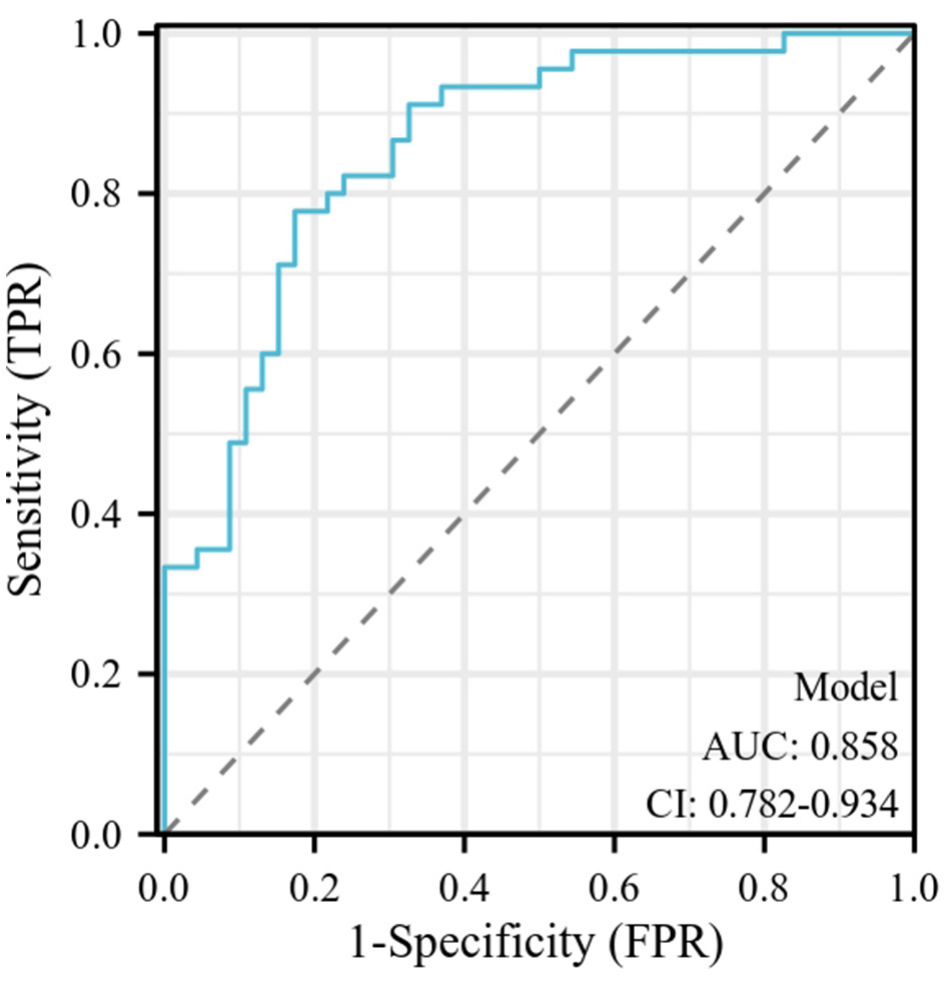
The ROC curve for predicting complications in infants with late-onset GBS sepsis. The ROC curve for predicting complications in infants with late-onset GBS sepsis was constructed using a combination of high PELOD-2 score, high pSOFA score, high creatinine levels, and hypoalbuminemia. The results showed that the optimal cut-off value for predicting complications was 0.021, with an AUC of 0.858 (95% CI: 0.782–0.934). GBS, Group B Streptococcal; PELOD-2, Pediatric Logistic Organ Dysfunction Score 2; pSOFA, Pediatric Sequential Organ Failure Assessment.

The ROC curve (Figure 1) for predicting complications in infants with late-onset GBS sepsis was constructed using a combination of high PELOD-2 score, high pSOFA score, high creatinine levels, and hypoalbuminemia. The results showed that the optimal cut-off value for predicting complications was 0.021, with an AUC of 0.858 (95% CI: 0.782–0.934), a sensitivity of 77.78%, and a specificity of 82.61%. The overall predictive accuracy was 80.22%, the positive predictive value was 81.40%, the negative predictive value was 79.17%, and the Youden index was 0.604. [Note: The ROC curve analysis for serum creatinine levels identified an optimal cut-off value of 0.78 mg/dL for predicting complications in infants with late-onset GBS sepsis. This cut-off yielded an AUC of 0.710 (95% CI: 0.609–0.812), with a sensitivity of 71.67% and a specificity of 75.61%].

## 4 Discussion

This study retrospectively analyzed the clinical data of infants under 3 months of age with late-onset GBS sepsis who were admitted to our hospital between 2007 and 2022. We found that a high PELOD-2 score and a high pSOFA score were significantly associated with an increased risk of complications. Additionally, high creatinine levels and hypoalbuminemia emerged as critical clinical indicators during the course of the disease.

The PELOD-2 score takes into account five organ systems—neurological, cardiovascular, respiratory, renal, and hematologic—and is widely used to assess illness severity in critically ill children. Its effectiveness in evaluating the risk of sepsis and septic shock has been validated in multiple studies (11–13). Recent studies have demonstrated that the PELOD-2 score is closely associated with both mortality and the incidence of complications in critically ill children (14). This study found that infants with late-onset GBS sepsis who had higher PELOD-2 scores exhibited a significantly increased incidence of complications. Elevated PELOD-2 scores typically reflect the severity of multi-organ dysfunction, which may lead to serious complications. Therefore, in clinical practice, a high PELOD-2 score should be regarded as a critical warning indicator, warranting prompt, and aggressive intervention in the early stages of disease progression.

The pSOFA score is an important tool for evaluating organ dysfunction in critically ill pediatric patients (10). An elevated pSOFA score has been shown to be closely associated with disease progression and poor prognosis in this population (15, 16). Xiang et al. (17) demonstrated that the pSOFA score can effectively predict the severity of pediatric severe sepsis, with a score greater than 6 serving as the optimal threshold for predicting in-hospital mortality (sensitivity: 93.9%, specificity: 77.6%). Consistent with these findings, the present study also confirmed that a high pSOFA score is an independent risk factor for complications in late-onset GBS sepsis. In summary, the pSOFA score offers a rapid and effective means of identifying infants with GBS sepsis who have significant organ dysfunction, thus providing a critical reference for timely and targeted clinical management.

In this study, elevated serum creatinine levels were identified as an independent risk factor for the development of complications in infants with late-onset GBS sepsis. Increased creatinine concentrations generally reflect a reduction in glomerular filtration rate, suggesting that systemic inflammatory responses may contribute to renal hypoperfusion. GBS infection can induce the release of bacterial exotoxins and trigger host immune responses, leading to microcirculatory disturbances, excessive release of inflammatory mediators, and tissue hypoperfusion, which together result in multiple organ dysfunction—with the kidneys being particularly vulnerable (18). Previous studies have demonstrated that renal impairment in severe sepsis or neonatal sepsis is closely associated with poor outcomes, possibly through exacerbating metabolic disorders, impairing toxin clearance, and disturbing fluid homeostasis, thereby promoting the progression of multisystem (19, 20). Therefore, dynamic monitoring of serum creatinine levels in infants with late-onset GBS sepsis is of great clinical importance, as it may serve as an early indicator for identifying the risk of complications and guide fluid management and renal protection strategies.

Hypoalbuminemia is a common clinical manifestation in infants with late-onset GBS sepsis. Albumin plays a vital role in maintaining vascular permeability, modulating immune function, and preventing fluid extravasation. In the present study, the incidence of hypoalbuminemia was significantly higher in the complication group among infants with late-onset GBS sepsis. Multivariate logistic regression analysis further identified hypoalbuminemia as an independent risk factor for complications in this population. Previous studies have demonstrated a close association between hypoalbuminemia and the severity of infectious diseases (21, 22). During severe infections, inflammatory mediators such as interleukin-1 (IL-1), interleukin-6 (IL-6), and tumor necrosis factor-alpha (TNF-α) can suppress hepatic albumin synthesis and accelerate its catabolism. In addition, increased vascular permeability leads to albumin leakage into the interstitial space, resulting in reduced plasma colloid osmotic pressure, tissue edema, and multi-organ dysfunction. Furthermore, decreased albumin levels impair the clearance of reactive oxygen species, exacerbating oxidative stress, and contributing to disease progression (9, 22). Therefore, for infants with late-onset GBS sepsis and concurrent hypoalbuminemia, early recognition and timely correction of hypoalbuminemia should be considered, alongside enhanced management of infectious complications, in order to improve clinical outcomes.

This study has several limitations that should be considered. As a retrospective, single-center analysis with a relatively small sample size, it is susceptible to potential selection bias. Although rigorous data selection and quality control were implemented, the findings require validation in large-scale, multicenter prospective cohorts. An important methodological constraint arises from the inherent delay in clinical diagnosis, which complicates the precise establishment of a temporal sequence between clinical variables and adverse outcomes, thereby introducing the possibility of reverse causality. Furthermore, while this study identified and analyzed clinical risk factors, it did not investigate the underlying biological mechanisms. Future research should, therefore, focus on elucidating the pathophysiological pathways linking these risk factors to disease progression and on evaluating the efficacy of various intervention strategies. Such studies are crucial to providing more precise and evidence-based management for neonatal GBS sepsis.

## 5 Conclusion

This study provides a theoretical basis for the individualized management of infants with late-onset GBS sepsis, with particular relevance to the early clinical identification of high-risk patients. We propose that the PELOD-2 and pSOFA scores may serve as valuable tools for prognostic assessment in this population. Notably, elevated scores should prompt intensified monitoring and timely intervention to prevent the development of complications. In addition, the presence of high creatinine levels and hypoalbuminemia should draw heightened clinical attention, as these factors may indicate increased disease severity. Early recognition and appropriate management of these conditions are essential to reduce the risk of adverse outcomes and improve the overall prognosis of affected infants.

## Data Availability

The original contributions presented in the study are included in the article/supplementary material, further inquiries can be directed to the corresponding authors.
